# Problematic Social Media Use Interventions for Mental Health Outcomes in Adolescents

**DOI:** 10.1007/s11920-025-01619-3

**Published:** 2025-07-15

**Authors:** Jason M. Nagata, Jacqueline O. Hur, Jonanne Talebloo, Seohyeong Lee, William W. Choi, Sean J. Kim, Jason M. Lavender, Megan A. Moreno

**Affiliations:** 1 Department of Pediatrics, University of California, San Francisco, 550 16th Street, 4th Floor, Box 0503, San Francisco, CA 94143 USA; 2 Military Cardiovascular Outcomes Research Program (MiCOR), Department of Medicine, Uniformed Services University of the Health Sciences, Bethesda, MD USA; 3 The Metis Foundation, San Antonio, TX USA; 4 Department of Pediatrics, University of Wisconsin–Madison, Madison, WI USA

**Keywords:** Problematic Media Use, Mental Health, Youth, Adolescents, Social Media

## Abstract

**Purpose of the Review:**

To narratively review recent literature addressing interventions aimed at reducing detrimental mental health effects of problematic social media use (PSMU) among adolescents aged 10–20.

**Recent Findings:**

The narrative review identified varying strengths and weaknesses of different types of interventions, which included therapy-based interventions (i.e., cognitive dissonance, self-compassion, mindfulness), media literacy, and social media limits. Moreover, features varied across interventions (i.e., duration, peer involvement, delivery format, setting).

**Summary:**

Programs that teach adolescents cognitive or behavioral strategies to engage with social media in a healthy manner appear to be more effective at improving long-term well-being than interventions that completely restrict social media use or warn adolescents of its negative effects. Tailoring interventions to the specific contextual factors salient to an individual or group may maximize the effectiveness and long-term impact of reducing PMSU and improving overall well-being. Future research should focus on longitudinal data to evaluate sustained intervention impacts.

## Introduction

Social media use is ubiquitous among adolescents and is often pervasive across multiple domains within an adolescent’s life, including community building, education, and self-exploration. However, the prevalence of social media engagement in this population raises concern for problematic social media use (PSMU), which is characterized by concerning behavior surrounding social media engagement and outcomes such as mood modification, tolerance, and interference with daily functioning [1]. The Surgeon General’s Advisory on Social Media and Youth Mental Health warns of the risks of social media and calls for urgent action, citing an alarming increase in social media use among adolescents in 2024, with up to 95% of 13- to 17-year-olds reporting social media use, and one-third on it almost constantly [2, 3]. As such, the influence of PSMU on psychosocial functioning and well-being in adolescents represents an important area of ongoing investigation.

Several systematic reviews detail the associations of PSMU with adolescent mental health concerns, including depressive symptoms, anxiety, and eating disorders [4, 5]. Social media engagement has also been found to contribute to negative body image in the form of facial dissatisfaction, self-objectification, and shame [6]. Notably, data indicate that adolescents themselves are aware of the potentially harmful mental health effects of social media use [7]. More broadly, problematic internet use (PIU), encompassing a broader spectrum of internet usage, has also been associated with adverse mental health outcomes in adolescents [8]. Multiple reviews have focused on existing interventions for PIU, with evidence suggesting the potential efficacy of certain psychological and behavioral approaches such as cognitive-behavioral-based interventions [9].

Though there has been a systematic review of the interventions for PSMU on the mental well-being of adults, there is a gap in the literature regarding this issue for adolescents [10]. The challenges of investigating links between social media use and mental health in youth, as well as developing and testing interventions, have also been highlighted [11]. Social media use often increases in adolescence and establishes patterns for lifelong habits and behaviors, thus effective interventions for PSMU in this population are crucial for mitigating short- and long-term adverse consequences. This narrative review, focused on recent literature, aims to identify and describe interventions addressing PSMU and its impacts on mental health outcomes among adolescents aged 10–20. This narrative review first identifies types of interventions addressed in recent studies and then discusses considerations regarding treatment delivery. The findings from this review may be relevant for clinicians, including mental health providers, working with adolescents who may exhibit PSMU.

## Types of Interventions

Table 1 reports examples of studies from the recent literature that address interventions targeting PSMU and/or its adverse impacts on mental health outcomes in adolescents aged 10–20. Table 2 summarizes the types of PSMU interventions identified and considerations regarding their delivery. For each intervention identified, we outline strengths and limitations noted from the original studies.

**Table 1:** Example recent publications on interventions for problematic social media use (PSMU) and mental health outcomes in adolescents

Author/ year	Sample (*n*)	Age range	Sex/Gender distribution (*n*)	Location	Intervention	Intervention provider	Control conditions	Key assessment measures	Mental health outcome
Bell et al. (2022)	290	12–13	151 female, 139 male	England	Digital Bodies Program (Therapy-based)	Mental health professionals	Before treatment	Body Image States Scale (BISS)	Body image satisfaction
Favini et al. (2023)	248	14–17	94 female, 154 male	Italy	Mindful Connections (Therapy-based Intervention)	Teachers	No treatment/ usual care	Bergen Social Media Addiction Scale (BSMAS)	Problematic social media use (PSMU)
Gordon et al. (2021)	487	11–15	245 female, 237 male, 5 non-binary	Australia	Social Media Literacy Program (Media Literacy)	Research assistants	Attention control	Visual Analogue Scales (VAS), Centre for Epidemiological Studies Depression Scale Revised (CESDR-10), Rosenberg Self-Esteem Scale (RSES)	Body image satisfaction, symptoms of depression, self-esteem
Kutok et al. (2021)	60	13–17	27 female, 21 male, 12 non-binary	USA	IMPACT Program Behavioral Challenge (Therapy-based Intervention)	Self-guided via app	No treatment/ usual care	WHO-5 Well-Being Index (WHO-5), PROMIS Psychological Stress (PROMIS-PS)	Well-being, psychological stress
Moreno et al. (2020)	1513	12–17	789 female, 724 male	USA	Family Media Plan (Social Media Limits)	School staff	Active control	Adolescents’ Digital Technology Interactions and Importance Scale (ADTI), Patient Health Questionnaire (PHQ-9)	Problematic internet use (PIU), symptoms of depression
Pietsch et al. (2022)	2,838	16–20	1,233 female, 1,605 male	Germany	14-Day Mobile App Challenge (Social Media Limits)	Teachers and mentors	No treatment/ usual care	Screen Time Reduction (STR)	Problematic social media use (PSMU)
Thai et al. (2021)	47	17–19	31 female, 16 male	Canada	Social Media Use Limitation (Social Media Limits)	Self-guided with email instructions	Comparative/ reference group	Body Esteem Scale for Adolescents and Adults (BESAA), Generalized Anxiety Disorder Scale (GAD-7)	Body image satisfaction, symptoms of anxiety
Weaver & Swank (2023)	54	14–19	22 female, 32 male	USA	Mindful Awareness Training (Therapy-based Intervention)	Educators and peer facilitators	Waitlist control	Social Media Use Questionnaire (SMUQ), Mindful Attention Awareness Scale - Adolescents (MAAS-A), Fear of Missing Out Scale (FoMOs)	Problematic social media use (PSMU), mindful attention, fear of missing out (FOMO)
Mahon & Harvey (2022)	102	15–17	54 girls, 48 boys	Ireland	Digital Social Media Adolescent Resilience Training (Therapy-based Intervention)	Primary Researchers certified in Compassionate Mind Training (CMT)	Waitlist control	Appearance Evaluation subscale of the Multidimensional Body-Self Relations Questionnaire (AE-MBSRQ), Body Appreciation Scale-2, Forms of Self-Criticizing/ attacking and Self-Reassuring Scale Short-Form (FSCRS-SF)	Average daily social media use, body (dis)satisfaction, body appreciation, self-criticism

**Table 2:** Summary of intervention types and delivery considerations for problematic social media use (PSMU)

Types of Intervention	Definition	Strengths	Limitations
Therapy-based	Incorporates elements of established therapeutic frameworks, such as cognitive behavioral therapy, mindfulness, and self-compassion, to help individuals recognize maladaptive thought patterns and behaviors	• Equips adolescents with practical coping strategies • Encourages self-regulation and intentional usage • Reshapes relationship with social media rather than removing it entirely	• Limited research on outcomes other than body image
Media literacy	Develops skills to critically evaluate and analyze information in the media	• Trains individual to navigate through negative content by examining the meaning of media	• Limited research • Minor intervention effects in younger populations
Social media limits	Limiting screen time on social media	• Efficient and direct method • Shown to be linked to an increase in life satisfaction and general well-being • Can be an effective method for encouraging engagement in other potentially more productive activities	• Blanket restriction without providing alternative coping strategies may not yield long-term success

Delivery considerations	Definition	Strengths	Limitations
Duration	The length of time over which interventions are implemented, including the number of sessions, frequency, and overall intervention period	Long duration: • More effective at fostering lasting changes in social media habits and the associated mental health outcomes	Long duration: • More difficult to implement in school or community settings • May have higher dropout rates and lower engagement over time
		Short duration: • Easy implementation • Low commitment for participant, which may contribute to higher participation rates	Short duration: • Potential for a decrease in sustained behavior changes due to a lack of reinforcement
Peer involvement	The degree of interaction or collaboration among adolescents during an intervention	High peer involvement: • Enhanced motivation and encouraged mutual accountability • Gives participants a chance to learn from one another’s experiences • Normalizes mental health discussion • Creates peer support networks	High peer involvement: • Environment may not be conducive to discussing sensitive issues due to fear of peer judgment • Limited participation among male students
		Low peer involvement: • Focus on individualized learning and can be self-paced • Less social comparison • Less distraction by peers	Low peer involvement: • Lack of external motivation and support
Administration	Administrative differences due to instructors having various backgrounds and different levels of training	Administration by teachers: • Existing relationship between teachers and students may enhance the effectiveness of the interventions • Teachers may have familiarity with the specific needs of their students, leading to better tailored or delivered content	Administration by teachers: • Lack of standardization
		Administration by trained facilitator: • Standardized training ensures that the interventions’ contents are delivered accurately and precisely across classrooms	Administration by trained facilitator: • May be more difficult to effectively engage students
School-based interventions	In-person, school-based intervention	• Particularly effective among early adolescents • Reduces socioeconomic barriers associated with seeking treatment outside of school to reach broad a number of adolescents	• Time and curriculum constraints may limit implementation feasibility
Virtual interventions	Virtual, app-based intervention	• Cost-effective and scalable alternative to in-person interventions • Teaches strategies in real-time by addressing PSMU in the context in which it occurs	• High attrition and low retention rate • May not be as tailored or interactive

### Therapy-based Interventions

Therapy-based interventions have emerged as a promising approach to addressing PSMU among adolescents. These interventions often incorporate established therapeutic frameworks such as cognitive behavioral therapy (CBT), mindfulness-based interventions (MBIs), and self-compassion-based strategies. Rather than framing social media as inherently harmful, therapy-based approaches seek to cultivate healthier engagement with social media by teaching adolescents to replace maladaptive habits with structured goals, positive thinking, and improved self-regulation [10].

One particularly effective therapy-based approach is mindfulness. MBIs, which have been developed to address a variety of psychological and behavioral conditions, broadly seek to cultivate open and non-judgmental attention and awareness to present moment experiences including thoughts, emotions, and sensations [12–14]. Specific strategies implemented within MBIs vary widely and can include breathing exercises, meditation, and grounding practices. Within the context of addressing PSMU, MBIs encourage the user to become intentional with their usage by monitoring and examining the motives behind their usage, potentially preventing escapism and absentmindedness when using social media [15]. MBIs have successfully reduced PSMU among adolescents, while also improving outcomes such as mindful attention [15]. Authors attributed this to the intervention’s ability to reinforce positive attitudes and promote self-regulation techniques rather than simply highlighting the negative consequences of PSMU. However, the extent to which this translates into downstream mental health outcomes such as body image, depression, or anxiety remains unclear, providing a potential direction for future research.

Dissonance-based interventions (DBIs) have also been examined in the context of PSMU. These interventions draw from cognitive dissonance theory by using psychological tension evoked by misaligned beliefs and behaviors to motivate cognitive change [16]. One study evaluating the efficacy of a DBI to improve adolescent body image, which involved deconstructing unrealistic appearance ideals and developing skills to challenge these ideals, found that it improved body satisfaction and thin-ideal internalization by promoting critical engagement with social media [17]. Specifically, by engendering cognitive dissonance related to social media and body image, this intervention encouraged adolescents to reshape social media behaviors to mitigate their harmful effects on mental health [17]. When combined with implementation intentions, or concrete and individualized behavioral plans to counteract compulsive social media use, DBIs showed long-term promise for improving certain adverse mental health outcomes of PSMU (i.e., body image concerns) by modifying beliefs to drive healthier behaviors.

Self-compassion-based interventions represent another approach that has been used to address PSMU and body image. Self-compassion practices encourage adolescents to respond to distress with empathy rather than self-judgment [18, 19]. One study examined a self-compassion-based intervention that helped adolescents recognize self-critical thoughts triggered by social media use and practice reframing these thoughts through breathing exercises and compassionate self-talk [19]. Participants reported significant improvements in body appreciation and satisfaction in both male and female populations, which were sustained at three-month follow-up [19].

Other approaches that have combined elements of cognitive-behavioral techniques have been similarly effective at improving PSMU and associated mental health outcomes. One intervention promoting self-regulation and intentional social media usage behaviors through perspective-taking exercises, self-monitoring tasks, and group discussions significantly reduced PSMU levels [20]. Another intervention employing cognitive restructuring, personalized goal setting, and emotional reflection observed significant improvements in overall mental well-being and reductions in psychological stress related to social media cyberbullying [20].

Together, therapy-based interventions that equip adolescents with psychological tools to understand, manage, and reshape their social media-related behaviors hold promise in buffering against the adverse effects of PSMU. This aligns with Plackett, Blyth, and Schartau’s systematic review conducted in adult populations, which found that therapy-based interventions were more effective due to their ability to foster beneficial relationships with social media rather than removing social media entirely [10].

### Media Literacy

Media literacy, broadly speaking, refers to skills to evaluate and analyze information gleaned from the media [21, 22]. Over time, this definition has expanded from traditional media sources to encompass social media and other multimedia content. Having the ability to critically consider and examine information from the media reduces the probability of falling victim to false narratives that digital content may portray [21, 22]. Rather than simply reducing screen time or unilaterally criticizing digital media, media literacy training helps the individual learn to critically maneuver through negative content. Despite its perceived benefits, research on media literacy and its effects on PSMU and mental health outcomes is limited, and studies that do delve into this topic report minor intervention effects between the control and experimental groups. For example, a recent study found that a social media literacy program had no effect on adolescent body image or depressive symptoms, with authors suggesting that awareness of social media’s harms does not necessarily reduce their negative mental health impacts [23]. Instead, authors posit that addressing the affective relationships that young people have with social media content may be more effective than solely employing rational, cognitive approaches [23]. Authors also noted that media literacy programs may be less effective for younger populations who may not yet possess the critical thinking skills needed to apply the intervention to real-life situations [23].

### Social Media Limits and Rules

Previous research has shown that simply limiting screen time on social media is linked to an increase in life satisfaction [24, 25] and other positive emotional outcomes such as appearance esteem among regular users [26]. Modalities for administering this treatment can vary, such as app-based screen limit interventions, which encourage reducing social media usage and have also been shown to improve general well-being [27]. Family-based interventions have also been examined, in which adolescents collaborate with family members to set media rules specific to their household [28]. In addition to their association with positive health outcomes, setting screen limits can be an effective method for encouraging engagement in other potentially more productive activities, such as hobbies and schoolwork [29, 30]. However, the effectiveness of such approaches may be compromised as social media becomes increasingly salient with age. Simply restricting access without providing alternative coping strategies may not yield long-term success, as Plackett, Blyth, and Schartau found social media limits to be the least effective in improving mental well-being among adults [10]. One study examining the effects of a Family Media Use Plan found no significant differences in adolescent media use, nor did they observe significant differences in sleep, physical activity, or depression post-intervention [28]. Researchers partially attributed this to increasing autonomy in adolescent participants, which may make them more resistant to externally imposed limits or rules around social media use [28].

## Delivery Considerations

### Duration

Duration refers to the length of time over which interventions are implemented, including the number of sessions, frequency, and overall intervention period. The effectiveness of interventions may be influenced by duration, as shorter programs tend to offer quick exposure, whereas longer programs have the potential to provide more sustained behavioral change. Different durations of intervention may cater to varying levels of engagement, retention, and accessibility. Studies implementing short-term interventions (4–5 sessions) showed some improvement in self-regulation and reduction in PSMU [15, 23]. Strengths included easier implementation and a low commitment for participants, which may have contributed to higher participation rates. On the other hand, there is a potential for a decrease in sustained behavior changes due to a lack of reinforcement. However, one single-session intervention addressing the impact of social media on adolescent body image was able to generate prolonged effects on body satisfaction. The researchers attribute this longevity to their use of implementation intentions, which directly train adolescents to apply what they learned to future situations [17]. Long-term interventions consisted of multi-month sessions with follow-up assessments [26, 27]. The repeated exposure and reinforcement tended to be more effective at creating lasting changes in social media habits and the associated mental health outcomes yet may be more difficult to implement in school or community settings [20]. Studies showed higher dropout rates and lower engagement over time that could be attributed to extended commitment [27].

### Peer Involvement

Interventions have been examined at different levels of peer involvement, which refers to the degree of interaction or collaboration among adolescents during an intervention. The degree of peer involvement has the potential to affect motivation or reinforcement of positive behaviors. Specifically, low peer involvement interventions focus on individualized learning and may benefit from the absence of social comparison or distraction by peers [26, 28]. In contrast, high peer involvement interventions may benefit from participants experiencing enhanced motivation and mutual accountability [31]. Involving peers may foster a supportive environment through group discussions, role-playing, and guided activities, giving participants the chance to learn from one another’s experiences with social media challenges [17]. Moreover, these group formats can normalize mental health discussions and create peer support networks that may help address PSMU. However, one recent study examining the effects of a group-based intervention received feedback from participants that the environment was not conducive to discussing sensitive issues due to fear of peer judgment. This limited participation among male students, potentially due to increased stigma surrounding discussions of male mental health [19]. Conversely, another study aimed at preventing and reducing the effects of cyberbullying found that lower peer involvement successfully improved participant well-being and stress levels while also being perceived positively by participants due to increased privacy [20]. These findings demonstrate that addressing issues with a greater degree of stigma, such as cyberbullying or body image, may benefit from less peer involvement. Given that each individual may respond differentially to low or high peer involvement, it may also be useful to assess their preferences prior to matching them with an intervention based on their particular needs and wants.

### Administration

The various interventions described in this review were administered by instructors of various backgrounds and different levels of training. School-based interventions commonly have teachers facilitate the intervention, as teachers can positively influence implementation and can motivate students to actively partake in the intervention [27]. The existing relationships between teachers and students may further improve intervention effectiveness. Teachers’ familiarity with the specific needs of their students may allow them to deliver content in a way that resonates with them, potentially leading to longer-lasting outcomes [23]. Other school-based interventions train facilitators who then follow a manualized approach, ensuring that the intervention’s contents are delivered accurately and consistently across classrooms [15]. However, external facilitators may struggle to engage students as effectively as teachers, which could hinder their ability to translate the skills learned from the intervention into real-life situations [12].

### School-based Interventions

Interventions for PSMU have been examined in both in-person and virtual contexts. Given that adolescence represents a sensitive period for behavior formation, in-person, school-based interventions provide access to a critical developmental window by targeting one of the most significant environments for adolescents [15, 31]. A review of interventions aimed at improving body image found that school-based interventions were particularly effective among early adolescents [23]. By reaching a broad number of adolescents, schools effectively facilitate primary interventions that encourage the formation of healthy media habits before problematic usage occurs, while reducing socioeconomic barriers associated with seeking treatment outside of school settings [23, 27, 31]. Despite this, time and curriculum constraints may limit the implementation feasibility of school-based interventions [20]. Policy changes that integrate social media use into the curriculum may also increase the feasibility of these interventions. For example, the UK now mandates the inclusion of information on social media and body image in health education curricula [17].

### Virtual Interventions

Virtual interventions offer a cost-effective and scalable alternative to in-person interventions [20, 26, 27]. By addressing PSMU in the context in which it occurs, adolescents can learn self-regulation and coping strategies in real-time [23]. App-based interventions are also capable of adhering to pandemic social distancing regulations, which is relevant given that many adolescents reported increased social media use and worsened mental health during the COVID-19 pandemic [20]. However, virtual interventions rely on user engagement, such as regularly checking notifications [20]. Many studies of web-based interventions report higher attrition rates and lower retention rates [27, 28]. One study evaluating an online Family Media Use plan found no significant differences in adolescent social media use, nor did they observe any differences in sleep, physical activity, or depression [28]. Researchers attributed this ineffectiveness to its virtual format, suggesting that the lack of reminders or reinforcement was not powerful enough to enact lasting behavioral change [28]. However, more interactive approaches, such as tailored app-based daily messages, have demonstrated higher acceptability and retention [20]. Given the merits and drawbacks of in-person and virtual interventions, future research could investigate the efficacy of hybrid approaches integrating school-based education with digital reinforcement strategies to maximize engagement and implementation feasibility.

## Conclusion

This narrative review highlights PSMU intervention strategies and associated delivery considerations from the recent literature. Overall, interventions that equip adolescents with concrete strategies for healthier engagement with social media have shown greater potential in improving long-term well-being. Media literacy programs, while effective in raising awareness of PSMU and potentially useful for prevention efforts, may lack the depth and individually tailored approach necessary to treat the mental health outcomes of PSMU. Similarly, social media limits and restrictions, despite their short-term benefits, fail to offer alternatives to generate sustainable results. Alternatively, therapy-based approaches may be more effective by using practical exercises and activities that promote the development of healthy behaviors with social media. These interventions also reinforce positive thought patterns and well-being beyond social media usage to address mental health outcomes arising from PSMU, such as body image. For long-lasting change, interventions should go beyond raising awareness of PSMU’s detrimental effects by actively equipping adolescents with alternatives and practical tools that will help them build sustainable habits, attitudes, and relationships with social media.

Despite these promising findings, there remains a significant gap in research regarding the long-term efficacy and accessibility of these interventions. Future research should prioritize longitudinal studies to assess the sustained impact of interventions on adolescent mental health and social media behaviors. Additionally, policy-oriented research could investigate ways to integrate social media education into school curricula and public health frameworks. Advancing our understanding of evidence-based interventions will help inform collaborative efforts between clinicians, educators, parents, and policymakers to empower adolescents in fostering healthy social media habits that protect their mental health.

## Key References

- Plackett R, Blyth A, Schartau P. The impact of social media use interventions on mental well-being: systematic review. J Med Internet Res. 2023;25:e44922.

**A systematic review evaluating social media use interventions aimed at improving mental health in adults.**

- Weaver JL, Swank JM. A mindfulness-based intervention for adolescent social media users: a quasi-experimental study. Journal of Child and Adolescent Counseling. 2024;10:3–14.

**Evaluates a mindfulness-based intervention aimed at reducing problematic social media use and improving life satisfaction among adolescents.**

- Bell BT, Taylor C, Paddock D, Bates A. Digital bodies: a controlled evaluation of a brief classroom-based intervention for reducing negative body image among adolescents in the digital age. Br J Educ Psychol. 2022;92:280–98.

**Evaluates a single-session CBT-based intervention aimed at improving adolescent body satisfaction, appearance ideal internalization, and self-objectification.**

- Mahon C, Hevey D. Pilot trial of a self-compassion intervention to address adolescents’ social media-related body image concerns. Clin Child Psychol Psychiatry. 2023;28:307–22.

**Examines the effects of a self-compassion intervention on improving social media-related body image concerns in adolescents.**

- Kutok ER, Dunsiger S, Patena JV, Nugent NR, Riese A, Rosen RK, et al. A cyberbullying media-based prevention intervention for adolescents on Instagram: pilot randomized controlled trial. JMIR Ment Health. 2021;8:e26029.

**Evaluates the mental health impacts of an intervention combining cognitive-behavioral therapy and motivational interviewing techniques.**

- Pietsch B, Arnaud N, Lochbühler K, Rossa M, Kraus L, Gomes De Matos E, et al. Effects of an app-based intervention program to reduce substance use, gambling, and digital media use in adolescents and young adults: a multicenter, cluster-randomized controlled trial in vocational schools in Germany. IJERPH. 2023;20:1970.

**Assess the effects of app-based screen limits on social media, gaming, and substance use in adolescents.**

## Data Availability

No datasets were generated or analysed during the current study.
